# The effect of oligofructose-enriched inulin supplementation on gut microbiota, nutritional status and gastrointestinal symptoms in paediatric coeliac disease patients on a gluten-free diet: study protocol for a pilot randomized controlled trial

**DOI:** 10.1186/s12937-017-0268-z

**Published:** 2017-08-22

**Authors:** Urszula Krupa-Kozak, Natalia Drabińska, Elżbieta Jarocka-Cyrta

**Affiliations:** 1 Department of Chemistry and Biodynamics of Food, Institute of Animal Reproduction and Food Research, Polish Academy of Sciences, Tuwima 10 Str, 10-748 Olsztyn, Poland; 2 Department of Clinical Pediatrics, Faculty of Medical Science, University of Warmia & Mazury, Oczapowskiego 2, Str, 10-719 Olsztyn, Poland

**Keywords:** Coeliac disease, Prebiotic, Oligofructose-enriched inulin, Gluten-free diet, Supplementation, Gut microbiota

## Abstract

**Background:**

A lifelong gluten-free diet (GFD) is regarded as the only proven and accepted therapy for coeliac disease (CD). However, even patients who strictly follow a GFD often suffer from intestinal symptoms and malabsorption. Selective modulation of intestinal microbiota with prebiotics could remedy various symptoms associated with CD. The use of prebiotics in the treatment of intestinal diseases remains insufficiently investigated. To our knowledge, this study makes the first attempt to evaluate the effect of prebiotic supplementation on gastrointestinal symptoms and nutritional status of children with CD. We hypothesized that adherence to a GFD supplemented with oligofructose-enriched inulin (Synergy 1) would deliver health benefits to children suffering from CD without any side effects, and that it would alleviate intestinal inflammation, restore and stabilize gut microbial balance and reverse nutritional deficiencies through enhanced absorption of vitamins and minerals.

**Methods:**

A randomized, placebo-controlled clinical trial was designed to assess the impact of the Synergy 1 on paediatric CD patients following a GFD. We randomized 34 children diagnosed with CD into an intervention group receiving 10 g of the Synergy 1 supplement daily and a placebo group (receiving maltodextrin) during a 12-week nutritional intervention. Selected biochemical parameters, nutritional status and the characteristics of faecal bacteria will be determined in samples collected before and after the intervention. Analysis of vitamins and amino acids concentration in biological fluids will allow to assess the dietary intake of crucial nutrients. The compliance to a GFD will be confirmed by a Food Frequency Questionnaire (FFQ-6) and the analysis of serum anti-tissue transglutaminase and faecal gluten immunogenic peptides (GIP).

**Conclusion:**

The identification of the beneficial effects of the Synergy 1 supplement on children with CD could have important implications for nutritional recommendations for CD patients and for alleviating the harmful effects of the disease.

**Trial registration:**

ClinicalTrials.gov Registration Number: NCT03064997.

**Electronic supplementary material:**

The online version of this article (doi:10.1186/s12937-017-0268-z) contains supplementary material, which is available to authorized users.

## Background

Coeliac disease (CD) is a chronic inflammation of the intestinal mucosa resulting from an excessive immune response to a dietary gluten (mixture of proteins found in wheat, rye and barley) in genetically predisposed individuals, both children and adults [1]. The disease is relatively prevalent, and it affects approximately 1% of the general population, although the majority of cases remain undiagnosed [1]. In addition to the inflammation of the small intestine, CD can also be manifested by other symptoms affecting different organs or tissues [2, 3]. The classical form of the disease involving intestinal symptoms and malabsorption (diarrhoea, vomiting, abdominal pain) is more frequently diagnosed in children than adults. Unapparent manifestations of the disorder involving non-intestinal clinical symptoms such as anaemia, decreased bone mineral density and mental abnormalities are more common in older patients. Untreated CD could lead to chronic inflammation, malabsorption and nutritional deficiencies. A strict, lifelong gluten-free diet (GFD) which requires complete abstinence from foods and grains that contain gluten (wheat, rye, barley) is regarded as the only proven and accepted therapy for CD. Research indicates that rigorous observance of GFD alleviates gastrointestinal symptoms [4] and restores gut health in CD patients [5], however, compliance with GFD guidelines varies considerably from 80% to less than 40% [6]. Many CD patients who observe a strict GFD continue to experience symptoms of the disease. In 20–80% of patients following a GFD, the mucosal lesions (Marsh II and III) were reported [7] similar to intestinal epithelia impairment that is noted in newly diagnosed patients. In microbiological studies [8–10], the diversity of *Lactobacillus* and *Bifidobacterium* bacteria was reduced in CD patients, which is associated with intestinal dysbiosis. According to Wacklin et al. [11], intestinal dysbiosis is linked with persistent gastrointestinal symptoms in CD patients on a GFD. Iron deficiency and anaemia are also common complications in CD, and their prevalence ranges from 12% to 69% of newly diagnosed patients [12]. Lower bone mass density was determined in 74% of adult CD sufferers who stuck to a GFD for long periods of time [10]. Taking into consideration the abovementioned research results, it could be suggest that GFD alone may not effectively reverse the clinical symptoms and re-establish intestinal microbiota balance in CD patients, even if GFD is still considered the most rational treatment approach. For this reason, several new therapies have been proposed as an alternative to a GFD [13] however, studies assessing the efficacy of the new treatments are expensive and are still at the research stage. Meanwhile, prebiotics as naturally-occurred plant-derived compounds seem to be promising and safe additive to a GFD demonstrating the beneficial influence on human health.

Prebiotics selectively stimulate the growth and activity of potentially health-promoting strains of bacteria in the intestine, mainly *Bifidobacterium* and *Lactobacillus*. Many studies report positive prebiotic-induced changes in the microbial composition alongside the beneficial health effects however, only few studies investigated the impact of prebiotics on patients with intestinal inflammation. Research investigating the effectiveness of prebiotics in managing chronic intestinal diseases is still in its infancy, but prebiotics’ ability to regulate the activity of gut microbiota could be harnessed to remedy various symptoms associated with CD. Data from the in vitro studies and the animal model studies, yet promising but do not allow to formulate a final conclusion about the effects of prebiotics therefore, the evidence for such beneficial effects in human subjects is urgently needed. Only few pilot human studies concerning the impact of prebiotics on intestinal inflammation is available [14, 15]. Besides the fact that a limited number of such human studies have been performed, most of them have limitations as they investigated prebiotic effects in combination with the administration of other ingredients or did not include an appropriate control group. To fill this research gap, a randomized, placebo-controlled clinical trial was designed to assess the influence of the oligofructose-enriched inulin on paediatric CD patients following a GFD.

Based on the literature data, it could be hypothesized that the inclusion of prebiotics in GFD could be an easy to administer and cost-effective alternative treatment for CD. Balanced gut microbiota is a crucial determinant of health, and further research is needed to evaluate the safety and efficacy of prebiotic supplements for CD patients. By presenting this research protocol we are counting on a productive discussion concerning a GFD and the possible dietary methods of its modulation aimed to increase the effectiveness of GFD in CD. The initiated dialog between researchers of various field of interest, including food scientists, dieticians, and gastroenterologists might give the rationale for the further interdisciplinary research, concerning the modulation of gut microbiota and the intestinal functionality induced by nutrients and functional dietary ingredients, in the wide context of the novel dietary strategies in health and disease. The establishment of the cooperation with other research centres may possibly result in the extensive multi-centre research concerning this interesting but scantly investigated subject.

### Research objective and hypothesis

We hypothesized that adherence to a GFD supplemented with oligofructose-enriched inulin would deliver health benefits to children suffering from CD without any side effects, and that it would alleviate intestinal inflammation, restore and stabilize balance of a gut microbiota and reverse nutritional deficiencies through enhanced absorption of vitamins and minerals. The main aim of this clinical trial is to evaluate the impact of an oligofructose-enriched inulin (Synergy 1) on selected biochemical parameters and nutritional status of paediatric CD patients following a GFD. Attempts are also being made to determine the influence of the oligofructose-enriched inulin supplement on the quantitative composition and the activity of intestinal microbiota.

## Study design

**Fig. 1:**
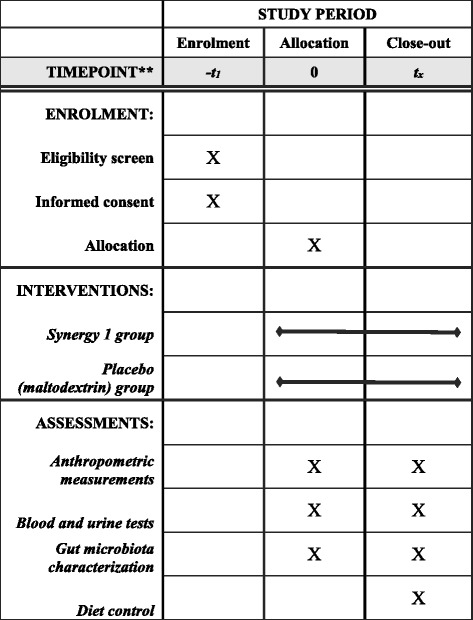
Content for the schedule of enrolment, interventions, and assessments according to SPIRIT requirements

**Fig. 2:**
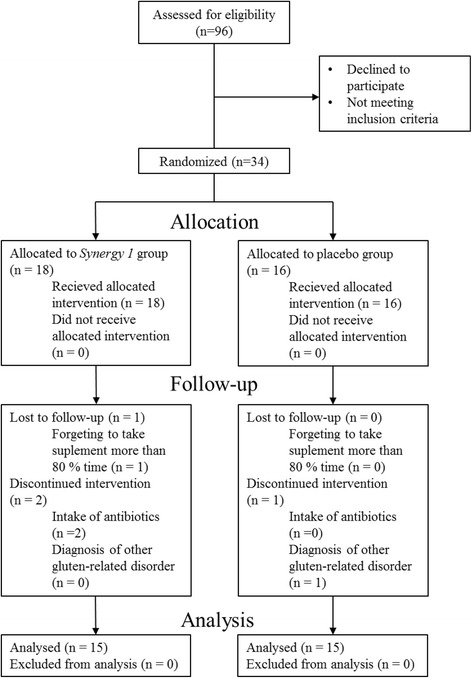
Flow diagram of participant recruitment during the trial according to CONSORT

This pilot, single-centre, randomized, parallel-group, placebo controlled nutritional intervention was conducted over a period of 12 weeks to determine the impact of oligofructose-enriched inulin on biochemical parameters, nutritional status and gut microbiota characteristics of paediatric CD patients who followed a GFD. The schedule of the enrolment, interventions, and assessments according to SPIRIT requirements is shown in Fig. 1. The clinical study design is presented in Fig. 2. This study is reported according to the Strengthening the Reporting of Observational Studies in Nutritional Epidemiology (STROBE-nut) checklist [16].

### Participant selection

Children and adolescents (aged 4 to 18 years) diagnosed with CD based on the criteria developed by the European Society for Paediatric Gastroenterology, Hepatology and Nutrition (ESPGHAN) [17] were recruited from among the patients of the Department of Paediatrics, Gastroenterology and Nutrition of the Children’s Hospital in Olsztyn at least 6 months before the study. Detailed inclusion and exclusion criteria are presented in Table 1. Around 150 children receive treatment for CD at the Department of Paediatrics, Gastroenterology and Nutrition of the Children’s Hospital in Olsztyn. The participants were recruited for the study between November 2015 and January 2016. A total of 96 CD patients were invited to participate in the pilot trial during regular visits at the clinic or by way of an invitation letter describing the details of the planned trial. Ultimately, 34 subjects who met the inclusion criteria (outlined in Table 1) and whose parents/legal guardians signed voluntary consent forms were enrolled in the study. The trial started in winter months, and all subjects were advised to continue their long-term vitamin D supplementation.

**Table 1:** Participant eligibility criteria

Inclusion criteria:
Coeliac disease confirmed by serological, genetic and biopsy analyses
Gluten-free diet for at least 6 months
Younger than 18
Patient of the Gastroenterology Clinic at the Regional Specialist Children’s Hospital in Olsztyn, Poland
Parents or caregivers are willing to give written informed consent for the child’s participation in the trial
Exclusion criteria:
Antibiotics in the month preceding the study
Medication for osteoporosis (bisphosphonates, calcium calcitonin), probiotics, prebiotics or fibre supplements
Poor or average overall health
Current enrolment in another research study
Recent surgery

### Ethical aspects

Parents and caregivers were informed about the potential benefits and risks of the dietary intervention, and they signed an informed consent form during the enrolment visit. The experimental design and procedures were approved by the Bioethics Committee of the Faculty of Medical Sciences of the University of Warmia and Mazury in Olsztyn (agreement No: 23/2015 of 16 June 2015). The study was registered at http://www.clinicaltrials.gov (registration number NCT03064997).

### Randomization and blinding

Patients have been randomly assigned to an intervention group (*n* = 18) or a placebo group (*n* = 16). Stratified randomization based on gender and age was conducted by a researcher not directly involved in the study. Separate randomization procedures were carried out for boys and for girls to produce comparable groups. The trial had a design where the researchers, laboratory personnel (excluding the researcher who distributed the supplement or the placebo to the participants) and the participants were not aware which subjects were receiving the supplement and which were receiving the placebo.

### Dietary intervention

The patients of the intervention group received oligofructose-enriched inulin (Orafti® Synergy 1, Beneo, Tienen, Belgium) for 3 months, whereas children of the placebo group received maltodextrin (Maltodextrin DE 20, Hotrimex, Konin, Poland) during the same period. Synergy 1 (DP3–9: 50% ±10, DP ≥ 10: 50% ±10, according to the producer) is the commercially available preparation of chicory inulin enriched by a specific fraction of oligofructose produced by partial enzymatic hydrolysis of chicory inulin. Both supplements, Synergy 1 and maltodextrin, were similarly light yellow powders of slightly sweet taste. Both substances are approved food grade ingredients, and they have been previously used in other clinical trials [18, 19]. Synergy 1 and maltodextrin were packed in identical white disposable sachets by the outsourcing company (DorTech, Pobiedziska, Poland). During the enrolment visit (T0), each participant was provided with a package of sachets containing the appropriate supplement in the amount required for a 3-month intervention. The participants from the experimental group received sachets containing 10 g of oligofructose-enriched inulin, whereas the participants from the placebo group received packages containing 7 g of maltodextrin. The packages with the appropriate supplement were distributed by a researcher who was familiar with the identity of the groups. The participants were instructed to consume the contents of one sachet daily for 3 months while closely following a GFD. The medium for suspending the supplement (water, juice or yoghurt) was chosen by the participants and could be changed during the trial. The participants were also provided with a questionnaire of compliance and adverse reaction, and were asked to record their daily intake of the supplement, any adverse reactions (gastrointestinal and other) and any other drugs taken during the trial. The participants who did not consume the supplement for more than 80% of the trial period were eliminated from the study. Before the end of the intervention, all participants were asked to complete a brief questionnaire assessing their mental wellbeing and stool characteristic (frequency and consistency). In the questionnaire, they were asked to provide information based on the observations made in the last week of the supplement intake. The participants were also asked to complete a Food Frequency Questionnaire with 6 answers (FFQ-6). At the end of the 12-week intervention, the participants were asked to return any remaining sachets of the Synergy 1 supplement or the placebo for assessment of the compliance.

### Data collection

Data were collected during the enrolment visit (T0), a visit at the end of the intervention (T1) and regular follow-up visits that took place every 3 months in the Gastroenterology Clinic. Demographic data were collected during the T0 visit. Anthropometric data and biological samples (blood, urine and faeces) were collected during T0 and T1 visits. The participants were provided with sterile containers for collecting urine and stool samples. An assessment of GFD compliance during the trial was performed at the end of the study.

### Anthropometric indices

Information about the participants’ body weight, height and nutritional status was collected by a nurse with the use of standard methods. The participants were weighed in light clothing and without shoes on a professional scale (Seca, Hamburg, Germany) with a weight limit of 200 kg and accuracy of 0.1 kg. Body height was measured on a standard stadiometer (Seca, Hamburg, Germany). The body mass index (BMI) was calculated as body mass divided by the square of body height, and it was expressed in kg/m^2^ and in standard deviation scores (SDS) based on age- and sex specific reference values. Detailed information relating to the measured anthropometric parameters is presented in Table 2.

**Table 2:** The participants’ baseline anthropometric data

	Total sample		Intervention group		Placebo group	
N	34		18		16	
Gender						
Girls	21 (61.8%)		11		10	
Boys	13 (38.2)		7		6	
	Range	Av^a^	Range	Av	Range	Av
Age (years)	4–17	10	5–17	10	4–16	10
Weight (kg)	15.0–78.0	35.3	15.0–67.9	36.6	16.3–66.8	33.7
Height (cm)	103–183	138.8	110–183	140.6	103.0–172.0	136.8
*BMI (kg/m* ^*2*^ *)*	12.5–28.4	17.1	13.5–23.3	17.2	13.7–28.4	17.0

^a^Average

### Diet control

In line with STROBE-nut recommendation [16], in the present study a modified FFQ-6 was applied [20]. It is a semi-qualitative food intake frequency questionnaire commonly used as a retrospective dietary assessment method suitable to evaluate the relation between consumed food and disease [21]. The applied FFQ-6 was a modified version from the original FFQ-6 (available online [20]) that was developed and validated using a reliability testing method in the population of young women (aging form 13–21 years old) from Olsztyn, Poland. The reliability of FFQ-6 was assessed by comparison of the results of first interview (test) with results obtained for the same group of respondents under the same conditions after 2 weeks (retest) [22]. The Spearman correlation coefficient and Cohen’s *kappa* coefficient tests were used to assess the reliability of the questionnaire FFQ-6 (test-retest). High Spearman correlation coefficient (ranged from 0.5 to 0.7) was determined for 42% of total food products, while very high correlation coefficient (0.7–0.9) was obtained for 50% of products. Cohen’s *kappa* coefficient ranged from 0.32 to 0.72. High test-retest reliability (*kappa* = 0.6–0.8) was obtained for 16% of total food products, while moderate reliability (*kappa* = 0.4–0.6) was obtained for 81% of products.

For the dietary assessment of a GFD compliance by CD children participating in the present study, we modified the original FFQ-6 by adding additional gluten-free items into the cereal products section [see Additional file 1], whereas other sections remain unchanged. At the moment the validation of modified FFQ-6 (by retest in 50% of study participants; 2-weeks after the test) studies take place. Modified FFQ-6 was distributed by mail to all study participants before T1 visit together with detailed instructions on how to complete it. The parents or caregivers were asked to fill in a questionnaire relating to the frequency with which different foods were consumed by the children during the entire trial (3-months intervention). The applied FFQ-6 reported the frequency of consumption of 63 food products representing eight groups: sweets and snacks; dairy products and eggs; cereal products; fats; fruits; vegetables; meat and fish products; beverages. The portion sizes were not considered. Consumption frequency was ranked on the following grading scale: never or almost never (1 point), once a month or less frequently (2 points), several times per month (3 points), several times per week (4 points), every day (5 points) and several times per day (6 points).

Moreover, to assess a compliance to a GFD, a serum anti-tissue transglutaminase (tTG) antibodies and faecal gluten immunogenic peptides (GIP) concentration will be analysed, whereas the balance of selected nutrients, in particular of vitamins and amino acids, will be analysed in plasma and urine of CD children at T0 and T1 visits.

### Sample collection

A fasting blood test was conducted in the morning, and blood samples were collected by a qualified nurse in the hospital laboratory. Three vials of blood were collected from every participant: 1) 1.6 mL into a vacuum tube containing EDTA for hematologic analysis; 2) 5 mL into a vacuum tube for biochemical serum analyses; 3) 5.5 mL into a vacuum tube containing an anticoagulant (heparin) for plasma analyses. Blood samples from vials 1 and 2 were processed according to standard procedures in the hospital laboratory. Blood samples from vial 3 were transported on ice, centrifuged at 3500 rpm for 10 min, and plasma aliquots (100 μL) were stored at −80 °C.

Not later than 3 days before the visit, faecal samples were collected, frozen and transported on ice to the clinic. On the day of the visit at the clinic, a fresh urine sample (second spontaneous urine) was collected in the morning (before breakfast). The samples provided by the participants were immediately separated into aliquots of approximately 1 mL (urine) and 100 mg (faeces) each and were stored at −80 °C until further analysis.

### Analysis of morphological and biochemical blood parameters

The complete blood count, blood biochemical parameters (creatinine, aspartate aminotransferase - AST, alanine aminotransferase - ALT, C-reactive protein – CRP, albumin, protein, calcium, phosphorus, magnesium, sodium, potassium, chloride, ferritin), tTG and urine biochemistry parameters (creatinine, calcium, phosphorus, magnesium, sodium, potassium) were analysed according to standard procedures in the Hospital’s Department of Laboratory Diagnostics and Transfusion Serology. The content of fat-soluble vitamins in plasma will be determined by a chromatographic method (HPLC-DAD for vitamins A and E) or in the enzyme-linked immunosorbent assay (ELISA) for vitamin D, using commercial kits. The content of water-soluble vitamins (B1, B2, B6, B9, B12) in plasma will be assayed using the HPLC-DAD method. The profile of amino acids in plasma will be analysed via EZ:Faast™ derivatisation method followed by gas chromatography/mass spectrometry detection (GC-MS). All parameters will be analysed at the beginning and at the end of the study.

### Urine analysis

Selected urine biochemical parameters (creatinine, calcium, phosphorus, magnesium, sodium, potassium) were analysed according to the standardized procedures in the diagnostic laboratory of the Children’s Hospital in Olsztyn. The analysis of amino acid profile in urine will be performed by the method described above (section: Analysis of morphological and biochemical blood parameters). All urine parameters were analysed both at T0 and T1 visits.

### Gut microbiota characteristics, short-chain fatty acids and GIP concentration

Bacterial genomic DNA will be isolated from faecal samples with the use of a dedicated commercial kit (GeneMATRIX Stool DNA Purification Kit, EURx, Gdańsk, Poland) for a quantitative analysis of gut microbiota. The quantitative characteristics of intestinal bacteria will be determined using the Real-Time PCR technique and group-specific primers. The DNA of strains representing the predominant bacterial groups will be the positive control, and it will be used to develop the standard curve. The total counts of *Bifidobacterium, Bacteroides*-*Prevotella*-*Porphyromonas, Clostridium coccoides, C. leptum* and *Lactobacillus* communities will be determined.

The concentration and profile of short-chain fatty acids (SCFAs), which are indicative of the activity of gut microbiota, will be determined in the faecal samples of all participants by gas chromatography with a flame ionization detector (GS-FID).

A quantity of GIP in faecal samples will be determined using a commercial Sandwich ELISA kit (Biomedal Diagnostics, Sevilla, Spain). Gut microbiota profiles, SCFAs and GIP concentration analysis will be performed in the faecal samples collected at T0 and T1 visits.

### Sample size

To our knowledge, this study makes the first attempt to evaluate the effects of fructan supplementation on children with CD, therefore, it was performed as a pilot trial without calculating sample size. Our plan was to recruit approximately 30 children (15 subjects per arm) with diagnosed CD on the assumption that the drop-out rate would reach approximately 20% (12 participants in each arm would complete the trial). The results obtained in the pilot study will be used to calculate the sample size for a fully-powered study that will be carried out in the future on paediatric CD patients receiving oligofructose-enriched inulin with GFD.

### Statistical analysis

Anthropometric and demographic data will be analysed using descriptive statistical methods. The participants whose reported intake of the Synergy 1 supplement or the placebo exceeded 80% of the prescribed intake will be included in final analyses. Statistical analyses will be performed in duplicate and expressed as mean values and standard deviation (SD). The influence of prebiotic supplemented-GFD and placebo supplemented –GFD on the measured parameters will be determined by one-way analysis of variance (ANOVA). If the differences between experimental and placebo groups are statistically significant (*P* < 0.05), the data will be subjected to post-hoc comparisons with the use of Fisher’s Least Significant Difference (LSD) test. The side effects of supplementation will be expressed by the percentage of each effect noted in the experimental group or the placebo group. The average consumption of food products by CD children during the intervention trial obtained from FFQ-6 will be compared in the Kruskal-Wallis test. Data will be processed statistically using Statistica 12 software (StatSoft, USA).

## Discussion

The use of prebiotics in the treatment of intestinal diseases has been poorly researched. To fill this research gap and cater to the growing demand for studies of the type, we proposed a complex interventional study investigating the impact of the Synergy 1 prebiotic supplement incorporated into a gluten-free diet on the biochemical parameters and the nutritional status of children with CD. According to the literature, prebiotics, in particular supplements combining short-chain and long-chain polymers, deliver beneficial effects for patients suffering from inflammatory bowel disease by inducing positive changes in the histology of the intestine (proliferation in crypts and Goblet cells, longer intestinal villi) and modulating endocrine and immune functions [23]. Prebiotics also have a great potential for improving and maintaining a healthy microbial balance in the intestinal lumen and on the surface of mucosa. Bifidobacteria and lactobacilli play a key role in this process. Healthy gut microbiota increases resistance to gastrointestinal infections, and it could also have immunomodulatory effects.

The described trial was inspired by the promising results of studies conducted on animal models as well as pioneering studies performed on human subjects. The results of several studies analysing the histological and biochemical parameters of animals with colitis suggest that prebiotics have anti-inflammatory properties. Prebiotic supplementation increased Bifidobacteria or Lactobacilli counts and, in selected studies, the concentration of butyrate in the intestines [24–26].

Despite the beneficial effects of prebiotics on animals, their usefulness in the treatment of inflammatory bowel disease in humans has been investigated by very few studies. Welters et al. [27] conducted a randomized, double-blind crossover study of patients with stable asymptomatic pouchitis whose diets were supplemented with inulin. The cited authors reported a decrease in pouchitis disease activity index (PDAI) scores as well as a decrease in gut pH, faecal *Bacteroides fragilis* counts and the content of secondary bile acids. In a different study, the administration of the Synergy 1 in combination with the *Bifidobacterium longum* probiotic to patients with ulcerative colitis significantly increased the counts of Bifidobacteria colonizing rectal mucosa [14]. The above was accompanied by a highly significant decrease in the levels of mucosal proinflammatory cytokines (TNF-α, IL-1a) and inducible β-defensins 2, 3 and 4 which are important markers of epithelial healing. Smecuol et al. [15] recently found that inulin-type fructans (ITF) contribute to the proliferation of *Bifidobacterium infantis* probiotic strains which have a confirmed beneficial effect on patients with active CD.

The described trial had certain limitations. Above all, sample size was not determined due to the absence of primary outcome data. However, the presented results can be used for retrospective calculations and to estimate the power of a full-size trial in the future. Another limitation is related to the participants’ characteristics, in particular the wide age span and differences in GFD duration. However, a protocol for reporting any effects observed in the group of available subjects was developed during this pilot trial. In a follow-up, full-size, multicentre study, a larger population will be investigated and differentiated according to age, duration of GFD and/or other parameters.

Despite these limitations, the results of this study could contribute to the development of novel nutritional strategies for CD patients. The originality and novelty of the proposed trial rises from the fact that the influence of fructans-supplemented GFD in CD children will be analysed for the first time, therefore, it is relevant to scrutinize the impact of this dietary compound on a broad spectrum of biomarkers assessing several important health-associated aspects. If untreated or not properly treated, CD can provoke irreversible pathological changes that require costly and prolonged treatment and rehabilitation. Our findings could also have important implications for nutrition and supplementation guidelines for children with CD as well as protocols for nutritional management of CD which aim to alleviate the harmful effects of the disease.

## Additional file

Additional file 1: Comparison between original FFQ-6 and modified FFQ-6 (*translated from Polish*). (DOCX 12 kb)

## Abbreviations

ALT: Alanine aminotransferase
ANOVA: One-way analysis of variance
AST: Aspartate aminotransferase
BMI: Body mass index
CD: Coeliac disease
CONSORT: Consolidated Standards Of Reporting Trials
CRP: C-reactive protein
EDTA: Ethylenediaminetetraacetic acid
ELISA: Enzyme-linked immunosorbent assay
ESPGHAN: European Society for Paediatric Gastroenterology, Hepatology and Nutrition
FFQ: Frequently consumed foods questionnaire
GC-FID: Gas chromatography coupled to flame ionization detector
GC-MS: Gas chromatography coupled to mass spectrometry detector
GFD: Gluten-free diet
HPLC-DAD: High performance liquid chromatography coupled to diode array detector
IgA: Immunoglobulin A
IL-1a: Interleukin 1a
ITF: Inulin-type fructans
LSD: Least Significant Difference
PCR: Polymerase chain reaction
PDAI: Pouchitis disease activity index
SCFA: Short chain fatty acids
SD: Standard deviation
SDS: Standard deviation scores
TNF-α: Tumour necrosis factor α
tTG: Anti-tissue transglutaminase
