# Supplementary material for: The effect of oligofructose-enriched inulin supplementation on gut microbiota, nutritional status and gastrointestinal symptoms in paediatric coeliac disease patients on a gluten-free diet: study protocol for a pilot randomized controlled trial
Source: Nutr J. 2017 Aug 22;16:47. doi: 10.1186/s12937-017-0268-z (PMC5568318; doi:10.1186/s12937-017-0268-z)
Supplement: Additional file 1: — Comparison between original FFQ-6 and modified FFQ-6 (translated from Polish). (DOCX 12 kb) [file 12937_2017_268_MOESM1_ESM.docx]

Additional file 1. Comparison between original FFQ-6 and modified FFQ-6 (*translated from Polish*)

| Original FFQ-6 | FFQ-6 after modification |
| --- | --- |
| **Cereal products** | **Cereal products** |
| **Whole-grain breads**: rye bread, graham, wheat or rye bread with grains, pumpernickel, grahams, crisp bread | **Whole-grain gluten-free breads**: oat, corn, brown rice or pseudoceraels bread (eg. buckwheat, amaranth, teff, millet ect) containing soja, pumpkin seeds, sunflower seeds, poppy ect. |
| **Refined breads**: wheat, rye or wheat-rye bread, toast bread, rolls, croissants | **Refined gluten-free breads**: light gluten-free bread, gluten-free toast bread, gluten-free buns, rolls, croissants |
|  | **Gluten-free bread baked at home** |
|  | **Gluten-containing bread** |
| **Not-refined coarse grains:** pearl barley, whole-grain pasta, buckwheat groats, brown rice | **Not-refined gluten-free coarse grains:** buckwheat groats, brown rice |
| **Refined fine grains:** manna groats, pearl barley, pasta, white rice, rice flakes | **Refined gluten-free fine grains:**, white rice, rice flakes, millet groats |
|  | **Gluten-containing groats** |
